# Genome-Wide Identification of the Invertase Gene Family in *Populus*

**DOI:** 10.1371/journal.pone.0138540

**Published:** 2015-09-22

**Authors:** Zhong Chen, Kai Gao, Xiaoxing Su, Pian Rao, Xinmin An

**Affiliations:** 1 National Engineering Laboratory for Tree Breeding, College of Biological Sciences and Biotechnology, Beijing Forestry University, Beijing, China; 2 Key Laboratory of Genetics and Breeding in Forest Trees and Ornamental Plants of the Ministry of Education, College of Biological Sciences and Biotechnology, Beijing Forestry University, Beijing, China; 3 Beijing Berry Genomics Company Limited, Beijing, China; Zhejiang University, CHINA

## Abstract

Invertase plays a crucial role in carbohydrate partitioning and plant development as it catalyses the irreversible hydrolysis of sucrose into glucose and fructose. The invertase family in plants is composed of two sub-families: acid invertases, which are targeted to the cell wall and vacuole; and neutral/alkaline invertases, which function in the cytosol. In this study, 5 cell wall invertase genes (*PtCWINV1*-*5*), 3 vacuolar invertase genes (*PtVINV1*-*3*) and 16 neutral/alkaline invertase genes (*PtNINV1*-*16*) were identified in the *Populus* genome and found to be distributed on 14 chromosomes. A comprehensive analysis of poplar invertase genes was performed, including structures, chromosome location, phylogeny, evolutionary pattern and expression profiles. Phylogenetic analysis indicated that the two sub-families were both divided into two clades. Segmental duplication is contributed to neutral/alkaline sub-family expansion. Furthermore, the *Populus* invertase genes displayed differential expression in roots, stems, leaves, leaf buds and in response to salt/cold stress and pathogen infection. In addition, the analysis of enzyme activity and sugar content revealed that invertase genes play key roles in the sucrose metabolism of various tissues and organs in poplar. This work lays the foundation for future functional analysis of the invertase genes in *Populus* and other woody perennials.

## Introduction

In higher plants, carbon autotrophy is a prominent feature and sucrose is the major form of transported sugar [1]. Sucrose is synthesised in source leaves and translocated to non-photosynthetic sink tissues. This disaccharide and its cleavage products, glucose and fructose, play central roles in cell metabolism and plant growth and development [2]. Sucrose utilisation as a source of carbon and energy depends on its hydrolysis into hexoses; in plants this reaction is catalysed by enzymes: sucrose synthase (EC 2.4.1.13) and invertase (EC 3.2.1.26). Sucrose synthase catalyses the readily reversible hydrolysis of sucrose into UDP-glucose and fructose, whereas invertase is responsible for the irreversible cleavage of sucrose to glucose and fructose [3].

The invertase family is classified into two sub-families based on solubility, subcellular localisation, and pH-optimum, and includes three types of invertase isoenzymes: cell wall, vacuolar, and cytosolic invertases [1]. The acid invertase sub-family appears to be localised to either the cell wall or vacuole [4,5]. The neutral/alkaline invertase sub-family is usually targeted to the cytosol [6]. It is believed that the acid invertase sub-family arises from respiratory eukaryotes and aerobic bacteria [7], while the neutral/alkaline invertase sub-family is closely related to the cyanobacterial invertases [8]. The existence of these two gene sub-families reflects the hypothesised origin of green algae and of higher plants through an endosymbiotic event in which a cyanobacterial endosymbiont became incorporated into a non-photosynthetic, respiratory eukaryote [9].

Cell wall and vacuolar invertases share some enzymatic and biochemical properties and have some common molecular features; however, the biochemical properties of cytoplasmic invertases differ markedly from those of the acid invertases [3]. Invertase activity is regulated at both the gene expression and enzyme activity levels. Plant acid invertase genes are regulated by sugars, pathogen infection, wounding, osmoregulation, and cold. In addition, acid invertase activity can be modulated by other factors, such as sugars, gibberellic acids, auxins, abscisic acids, cytokinins, brassinosteroids, ethylene, and proteinaceous inhibitors [1,2]. Resulting from difficulties in purification and weak or unstable enzymatic activity, there’s a paucity of knowledge on neutral/alkaline invertases [10]. Despite this, neutral/alkaline invertase genes have been described in *Vitis vinifera* [11], *Oryza sativa* [6], sugarcane [12] and peaches [13]. Acid invertases can hydrolyse fructose-containing compounds aside from sucrose, such as raffinose and stachyose, and they are strongly inhibited by heavy metals; however, sucrose is the sole substrate of neutral/alkaline invertases, which are not restrained by heavy metals [1].

In this study, we performed a genome-wide identification and characterisation of invertase genes from *Populus* and revealed an invertase gene family with a total of 24 members according to the poplar genome sequence in Phytozome v. 9.1. The analysis in this work focused mainly on sequence phylogeny, gene structure, chromosomal location and expression profiles in various tissues, and responses to salt/cold stress conditions and pathogen infection. We also investigated invertase activity and sugar content (sucrose, glucose and fructose) in various tissues and organs of poplar. Our results provide a foundation for further studies to gain a comprehensive understanding of the physiological roles of invertase genes of *Populus* in the regulation of important biological processes.

## Materials and Methods

### Database search and sequence retrieval

*Arabidopsis thaliana* invertase gene sequences were obtained from The *Arabidopsis* Information Resource (TAIR10) [14]. To anchor the entire *Populus* invertase gene family, the amino acid sequences of *Arabidopsis* invertase members were used as a query in our BLAST search of the Joint Genome Institute (JGI) Phytozome portal [15]. This search enabled us to identify sequence similarities using the *Populus trichocarpa* genome data and gene annotation hosted in Phytozome v. 9.1. Bioinformatics analysis, such as composition, physical and chemical characterisation, and conserved functional domains of the invertase gene family were performed using the Expert Protein Analysis System (ExPASy).

### Gene structure and phylogenetic analyses

Genomic, transcript, CDS and protein sequences of the *Populus* invertase gene family members were downloaded from Phytozome v. 9.1. The exon/intron structure of individual genes was illustrated using the Gene Structure Display Server (GSDS) software [16] by alignment of the cDNAs with their corresponding genomic DNA sequences from Phytozome. Multiple alignments of full-length protein sequences were performed using ClustalX. The unrooted phylogenetic trees were constructed with MEGA v. 6.0 [17] using the neighbour-joining (N-J) method, poisson model, pairwise deletion method, and a bootstrap test with 1,000 replicates.

### Chromosomal location and gene duplication

The chromosomal location of each *Populus* invertase gene was determined using GBrowse based on chromosome information for *P*. *trichocarpa* provided in Phytozome. Identification of segmental duplications arising from the whole-genome duplication event in the Salicaceae (salicoid duplication) was accomplished as described previously [18]. The tandem gene duplications in *Populus* were identified according to the same criteria described for rice. Genes separated by five or fewer gene loci in a range of 100-kb distance were considered to be tandem duplicates [19].

MEGA v. 6.0 was used to form the pairwise alignments of the paralogous nucleotide sequences, with the corresponding protein sequences as the alignment guides. Ks (synonymous) and Ka (nonsynonymous) substitution rates were estimated using the CODEML software of PAML [20]. Divergence time (T) was calculated using a synonymous mutation rate of k substitutions per synonymous site per year as T = Ks/2λ (λ = 9.1 × 10^−9^ for *Populus*) [21].

### Plant materials and treatment

All plants in this study are grown and maintained in the nursery of Beijing Forestry University (Beijing, China). No specific permissions were required because this nursery is a place for teaching and scientific research. This study did not involve any endangered or protected species. Root, stem, and leaf samples were obtained from 1-month-old tissue-cultured plants of *P*. *tomentosa*. Latent leaf buds (December) and leaf buds in the germination phase (February of the following year) were collected from adult *P*. *tomentosa*. To examine salt stress, the roots of 1-month-old tissue-cultured plantlets were removed from the medium and then submerged in 400 mM NaCl [22], and the roots and leaves were harvested after 4 h. For cold stress, leaves of tissue-cultured plantlets were held at 4°C for 4 h and then collected. For the pathogen infection study, the main stems of *P*. *tomentosa* were obtained from adult trees and cut into small sections of 30 cm lengths. *Botryosphaeria dothidea* was cultured on potato dextrose agar (PDA) for 7 days at 25°C in the dark. For inoculation, three 5-mm-diameter wounds were created in the bark of each stem at 10-cm intervals with a hole punch. Five-millimeter plugs of PDA without (control) or with *B*. *dothidea* were placed in the wound, packed with moist and sterile cotton, and sealed with plastic wrap to prevent desiccation and contamination. Stems were maintained hydroponically at ~26°C under a 12/12-h light/dark cycle. After 2 weeks, bark and developing xylem tissue from both infected and uninfected stems were harvested [23]. Root, stem and leaf from a single plant served as a biological replicate and three biological replicates were collected for assays and stress treatments. Leaf buds were sampled from five individuals (biological replicates) and pooled to promote sample homogeneity and decrease sampling bias. All samples were frozen in liquid nitrogen and stored at -80°C until further use. Total RNAs of all samples were isolated as described previously [24].

### Quantitation of sugars and enzymatic assays

Sucrose, glucose and fructose contents were determined using a previously reported enzymatic method [25,26]. Sugar concentration was defined as mg·g^-1^ fresh weight (FW).

All enzyme extraction steps were performed at 4°C. Cell wall invertase, vacuolar invertase and neutral invertase were extracted according to the method of Li et al. [27] with some modifications. Frozen flesh (1.0 g) was ground to a fine powder in liquid nitrogen using a mortar and pestle, and then homogenized in 200 mM HEPES-KOH (pH 8.0) containing 5 mM MgCl_2_, 2 mM EDTA, 2.5 mM DTT, 1% (v/v) Triton X-100, 4% (w/v) PVPP, 0.1% (w/v) BSA, and 10% (v/v) glycerol. The extract was centrifuged at 12,000×*g* for 30 min at 4°C and immediately desalted in a PD-10 column equilibrated with 50 mM HEPES-KOH (pH 7.5) containing 10% glycerol, 5 mM MgCl_2_ and 1 mM EDTA. The eluate was assayed for VINV and NINV activity. For CWINV, the pellet was washed three times with the desalting buffer, and the protein bound to cell wall was separated by incubation in the extraction buffer with 1 mM NaCl added at 4°C overnight. Then, the extract was centrifuged and desalted as above.

CWINV and VINV activities were assayed by modifying a previously described method [26,28]. The reaction mixture, which contained 100 mM phosphate-citrate buffer (pH 4.8), 100 mM sucrose, and 200 ul of the desalted extract, was incubated for 30 min at 37°C, and terminated by boiling in water for 5 min before adding 0.75 M Tris-HCl buffer (pH 8.5). The assay process for NINV activity was the same except that 100 mM HEPES-NaOH (pH 7.5) was used to replace the phosphate-citrate buffer. Enzyme activity was defined as U·g^-1^ FW.

### Gene expression

Transcriptome sequencing (RNA-Seq) analysis was used to evaluate the expression of *P*. *tomentosa* invertase genes. RNAs were sequenced using Illumina paired-end technology and an Illumina HiSeq2000 platform. High throughput sequencing was performed at the Beijing Yuanquanyike Biological Technology Co., Ltd. (Beijing, China). Data processing and *de novo* assembly were described by Ye et al. [29] and Liao et al. [23]. Gene expression levels were estimated using the number of mapped reads per kilobase of the exon region per million mapped reads (RPKM) values computed as proposed by Mortazavi et al. [30].

## Results

### Identification of invertase genes in *Populus*

To identify *Populus* invertase genes, we conducted a BLASTP search against the *Populus* genome database (Phytozome v. 9.1) using known protein sequences of invertase genes from *Arabidopsis* as queries; the resulting sequences were used as secondary queries. By removing redundant sequences, 24 putative invertase genes (8 from the acid invertase sub-family and 16 from the neutral/alkaline invertase sub-family) were identified in the *Populus* genome. After manual reannotation and confirmation of the protein characteristic domain, the 24 *Populus* invertase genes were designated *PtrCWINV1*-*5*, *PtrVINV1*-*3*, and *PtrNINV1*-*16* following the nomenclature proposed in a previous study [6]. The information on poplar invertase genes in the latest database (Phytozome v. 9.1) varies considerably from that in previous genome database and assembly. Based on the transcript number of genes from this study, we increased the total number to 45 (20 in the acid invertase sub-family and 25 in the neutral/alkaline invertase subfamily). *PtrCWINV1* and *PtrNINV 2*–*6* had two transcripts, *PtrCWINV4*, *PtrVINV3*, *PtrNINV1* and *PtrNINV8* had three transcripts, while *PtrVINV2* had eight transcripts. It is worth noting that the sizes of the genomic DNA, transcripts, CDS, and the numbers of peptide residues, have also been updated, along with the theoretical Mw and pI and the location of the functional domains (S1 and S2 Tables). Furthermore, we identified invertase genes from 10 other plant species, including the dicotyledonous angiosperms *Medicago truncatula*, *V*. *vinifera*, *Malus* × *domestica*, *Glycine max* and *Citrus sinensis*, and the monocotyledonous angiosperms *O*. *sativa*, *Brachypodium distachyon*, *Sorghum bicolor* and *Zea mays*. All angiosperm genomes, as well as the *Physcomitrella patens* genome, contain invertase genes. The numbers of invertase genes identified in the 10 other plant species are shown in Table 1.

**Table 1:** Numbers of invertase genes within each plant species according to transcript data.

Species	Acid	Neutral/Alkaline
*P*. *trichocarpa*	20	25
*A*. *thaliana*	13	13
*M*. *truncatula*	12	7
*V*. *vinifera*	8	11
*M*. *domestica*	8	19
*G*. *max*	35	23
*C*. *sinensis*	20	20
*O*. *sativa*	16	14
*B*. *distachyon*	15	9
*S*. *bicolor*	15	9
*Z*. *mays*	21	19
*P*. *patens*	16	9

### Gene structure and phylogenetic analyses

**Fig 1:**
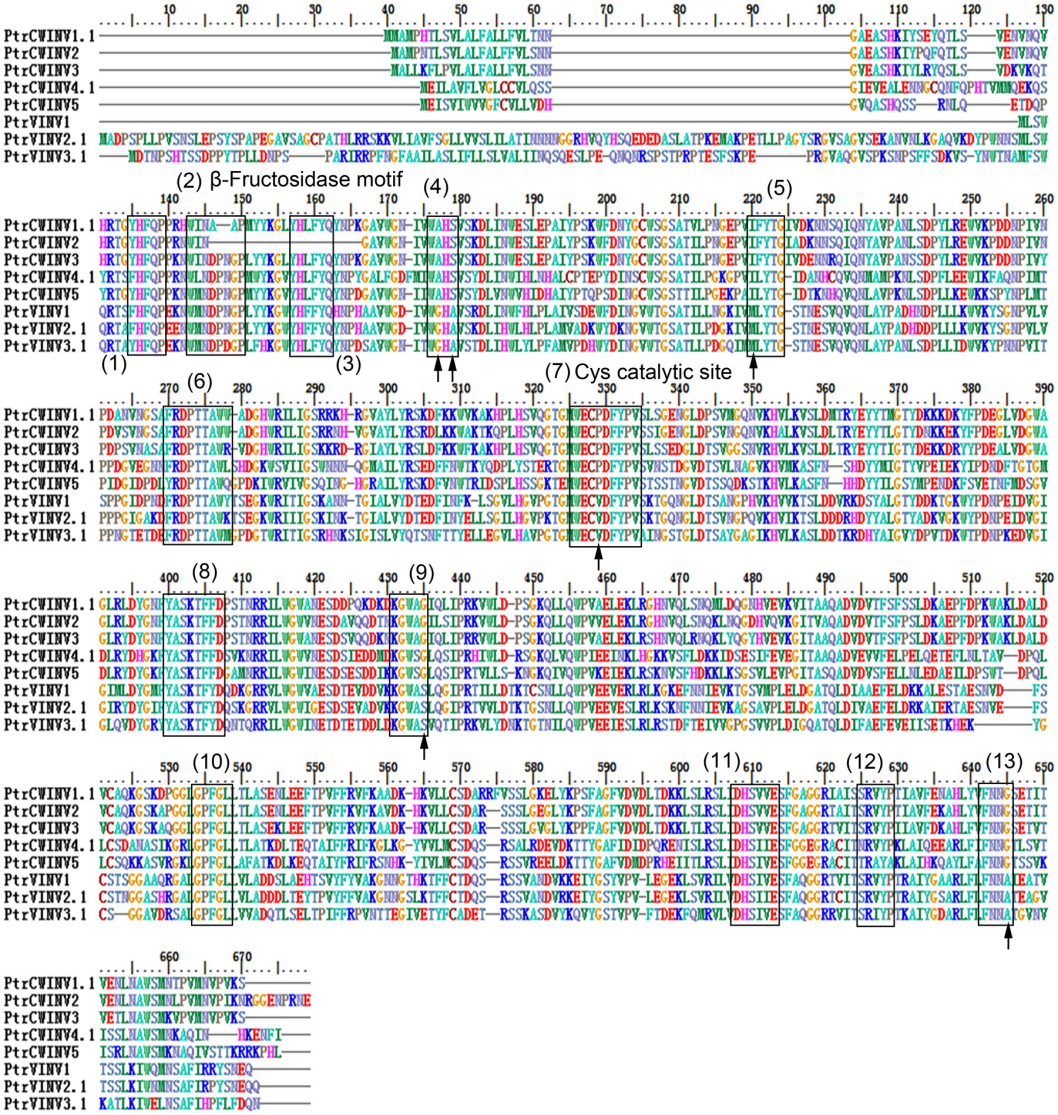
Multiple alignment of the acid invertase sub-family in *Populus*. The boxed region indicates the 13 well-conserved regions from known acid invertases of selected green plants. Arrows indicate the six amino acids that are consistently different between cell-wall and vacuolar invertases.

**Fig 2:**
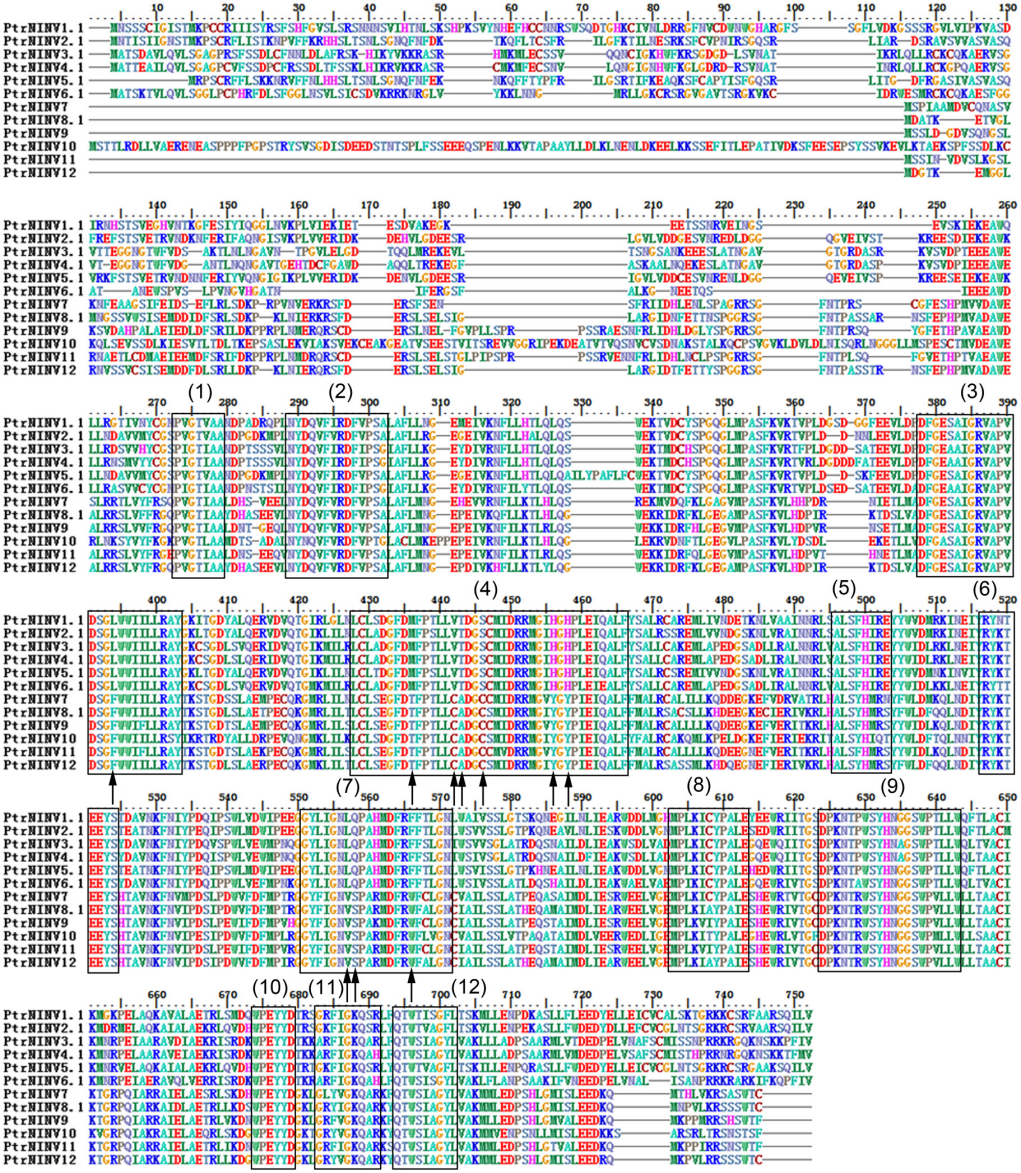
Multiple alignment of the neutral/alkaline invertase sub-family in *Populus*. The boxed region indicates the 12 well-conserved regions from known neutral/alkaline invertases of selected green plants. Arrows indicate the ten amino acids that are consistently different between the α and β clades.

**Fig 3:**
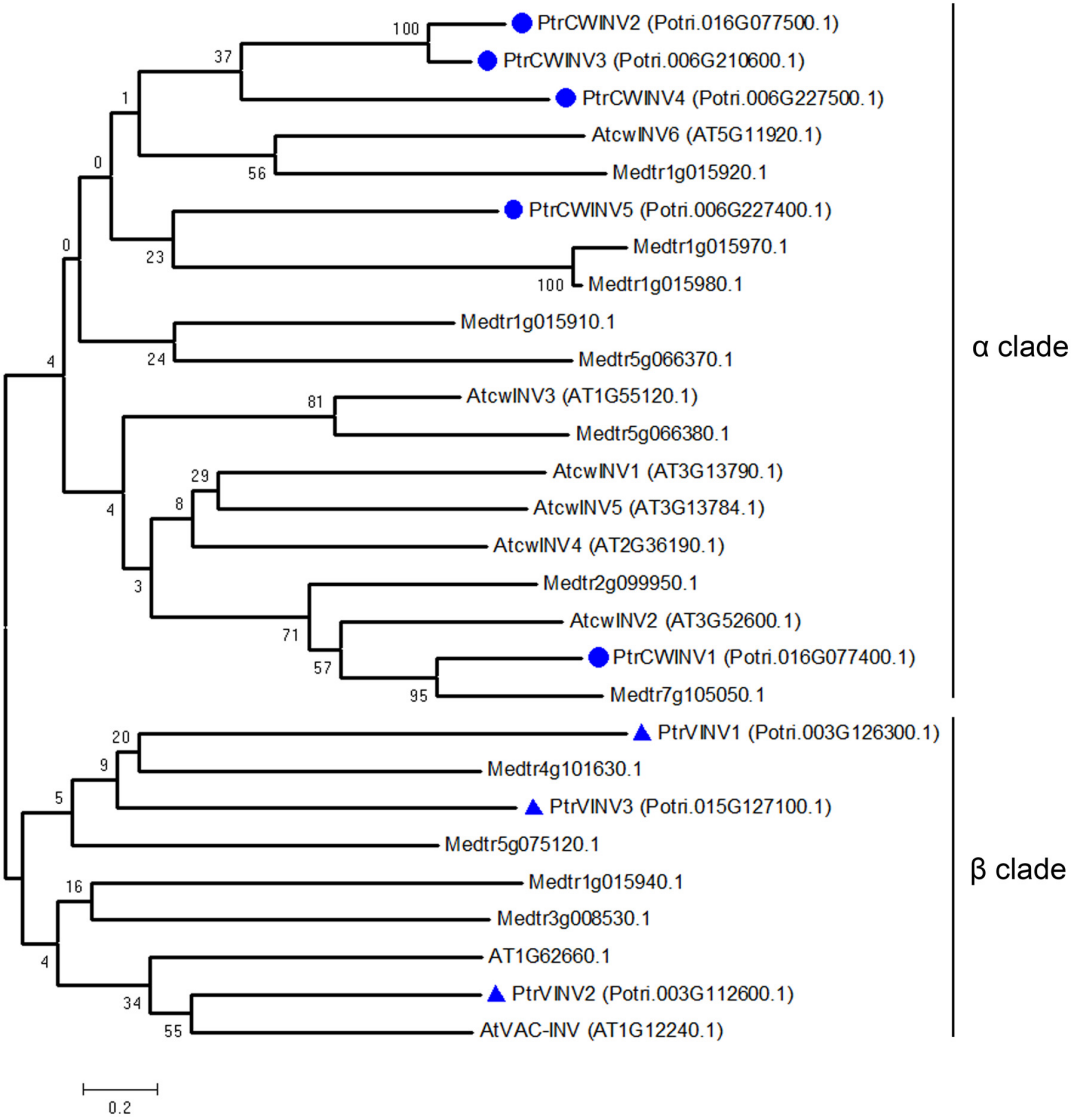
Phylogenetic tree of acid invertase proteins from *Populus*, *Arabidopsis* and Medicago. The α clade contains cell-wall invertases (*PtrCWINV1*-*5*) and the β clade contains vacuolar invertases (*PtrVINV1*-*3*).

**Fig 4:**
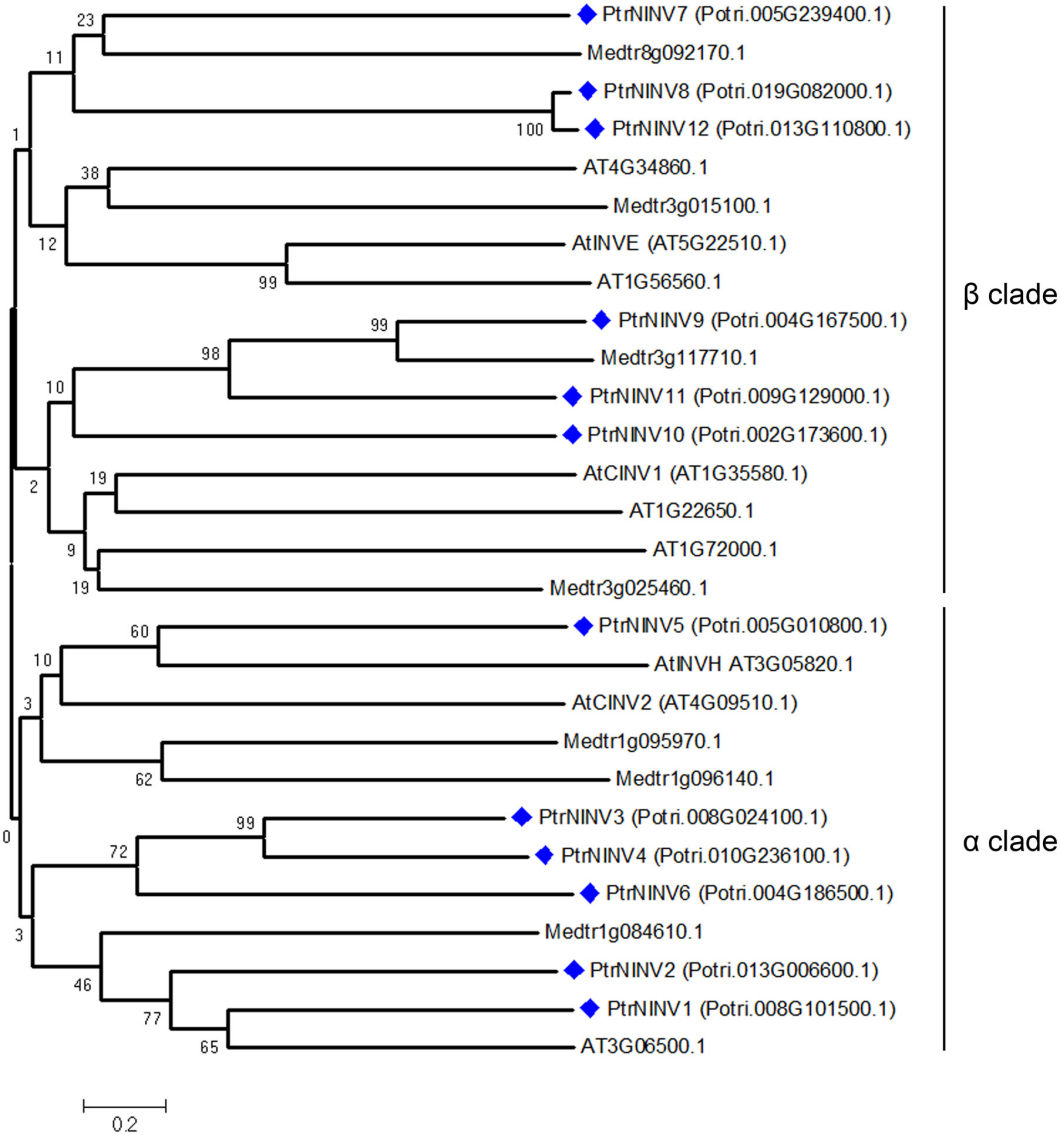
Phylogenetic tree of neutral/alkaline invertase proteins from *Populus*, *Arabidopsis* and Medicago. The α clade (*PtrNINV1*-*6*) and the β clade (*PtNINV7*-*12*) are indicated. *PtNINV13*-*16* are not included. Bootstrap values are generated as a percentage of 1000 repetitions.

**Fig 5:**
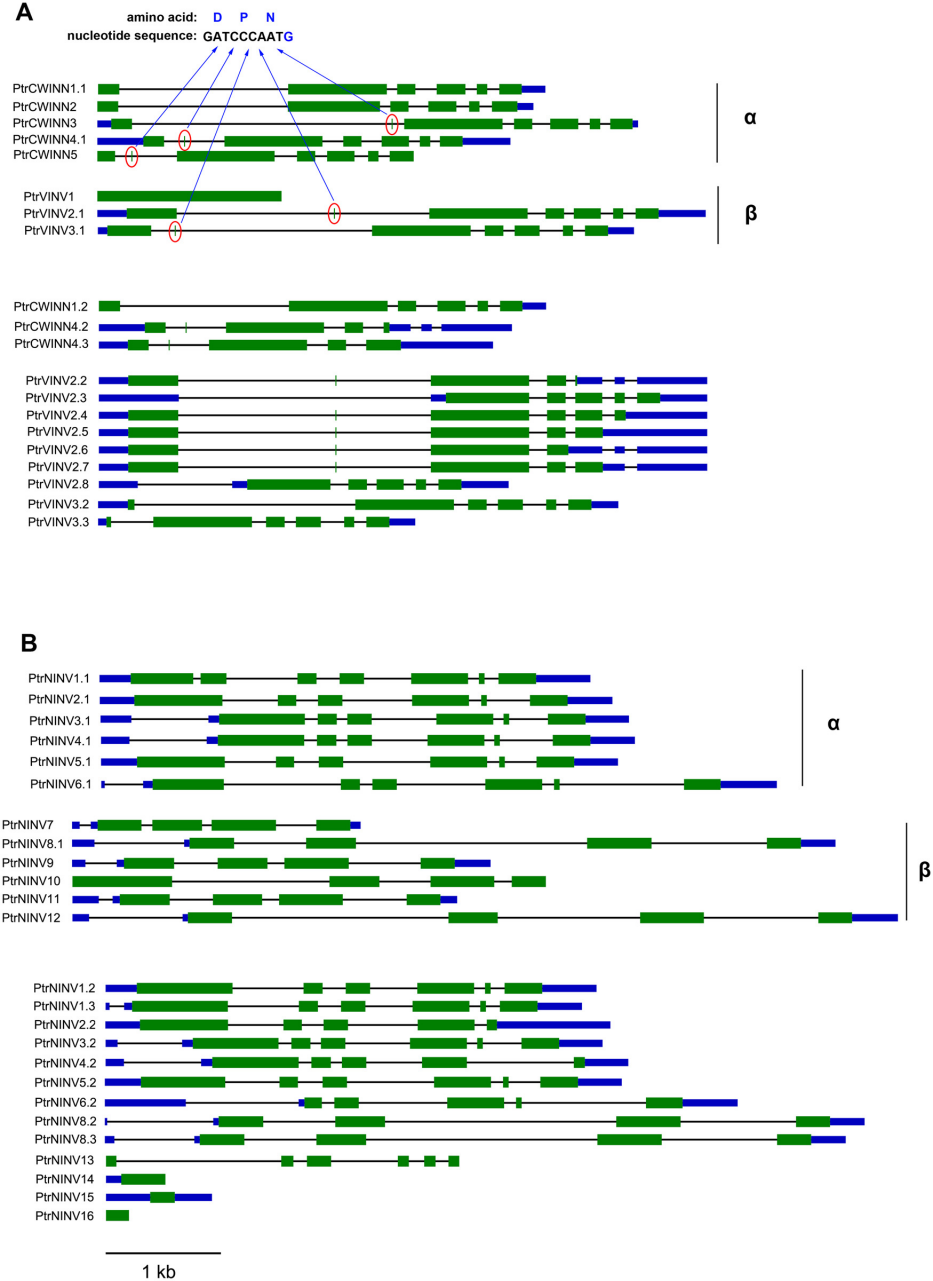
Schematic representation of the structure of the invertase gene family in *Populus*. Exons and untranslated regions (UTRs) are indicated by green and blue boxes, respectively, and black lines between boxes represent introns. **(A)** Acid invertase sub-family. The arrows indicate that a mini-exon contributes the tripeptide DPN. **(B)** Neutral/alkaline invertase sub-family.

To obtain further insight into the evolutionary history and distinct origin of the acid and neutral/alkaline invertase sub-families in *Populus*, we performed amino acid alignments (Figs 1 and 2) for the *Populus* invertase gene family, constructed a phylogenetic tree (Figs 3 and 4) using the full-length invertase protein sequences and compared the exon/intron organisation (Fig 5) of each individual gene. Phylogenetic analysis revealed that the acid invertase sub-family could be separated into two clades, α and β, one of which is inferred to be cell-wall-targeted while the latter vacuole-targeted (Fig 3). The neutral/alkaline invertase sub-family is also divided into two clades (α and β) (Fig 4), which are supported by the exon/intron structure (Fig 5B). A previous study reported 13 well-conserved regions in acid invertases and 12 conserved motifs in neutral/alkaline invertases in green plants [6]. In this study, 6 of the eight acid invertases, and all 12 of the neutral/alkaline invertases (PtrNINV1-12), were predicted to contain all of the conserved motifs (Figs 1 and 2; boxed). PtrCWINV1.1 and PtrCWINV2 had incomplete second conserved motifs, while PtrCWINV2 also lacked the third motif of acid invertases (Fig 1).

In the acid invertase sub-family, sequence comparison revealed that the genes share differential sequence identity at the nucleotide level (43.62% to 87.61%) within the coding region, and at the amino acid level (34.50% to 85.20%) (S3 Table). All eight genes contain the WECXDF motif; the exceptions being *PtrCWINV1*.*1* and *PtrCWINV2*, the other six genes contain the NDPNG motif. These two motifs are well-conserved in this family and are essential for catalytic activity [7]. Six amino acids in the conserved motifs consistently differed between the cell-wall (α clade) and vacuolar (β clade) invertases (Fig 1; arrows). Using numbering based on PtrVINV2.1, the residues are A177G, S179A, I220M, P329V, G435S and G645A. With the exception of *PtrCWINV1*, *2* and *PtrVINV1*, the acid invertase sub-family is encoded by seven exons (Fig 5A). *PtrVINV1* is a no-intron invertase that belongs to the β clade, presumably targeted to the vacuole (Figs 3 and 5A). Although this gene contains no introns, it retains all 13 well-conserved motifs related to the acid invertase sub-family (Fig 1).

In the neutral/alkaline invertase sub-family, sequence comparison revealed that the genes share different sequence identity at the nucleotide level (44.58% to 93.49%) within the coding region, and at the amino acid level (39.08% to 94.61%) (S4 Table). Four distinct gene pairs with high nucleotide (or amino acid) sequence identity were found between *PtrNINV2* and *PtrNINV5*, *PtrNINV3* and *PtrNINV4*, *PtrNINV8* and *PtrNINV12*, and *PtrNINV9* and *PtrNINV11*; their homologies ranged from 88.99% to 93.49% (86.85% to 94.61%) (S4 Table). The neutral/alkaline invertase sub-family was also divided into α and β clades (Fig 4) that varied consistently at 10 amino acid residues within the conserved motifs (Fig 2; arrows). Using numbering based on PtrNINV10, the residues were L394F, M436T, V442C, T443A, S446C, H456Y, H458Y, L557V, Q558S and F566W. The α clade contains *PtrNINV1*-*6*, encoded by six exons with conserved locations except for *PtrNINV1* (seven exons), whereas the β clade contains *PtrNINV7*-*12*, encoded by four exons (Fig 5B). The different exon/intron structures suggest that the two clades originate from distinct ancestral genes.

### Chromosomal location and gene duplication

*In silico* mapping of the gene loci showed that the 24 *Populus* invertase genes were distributed among 14 of the19 *Populus* chromosomes (Chr). Chr 6 and Chr 14 had the largest number, with three *Populus* invertase genes. In contrast, no *Populus* invertase genes were found on Chr 1, 7, 11, 12 and 18 (S1 Fig).

To determine the possible evolutionary relationship between the *Populus* neutral/alkaline invertase genes and potential segmental duplications, *Populus* neutral/alkaline invertase genes were mapped to the duplicated blocks established in a previous study. *PtNINV1*-*13* and *PtNINV16* (87.50%, 14 of 16 genes) were located within the duplicated regions, while only *PtrNINV14* and *15* were located outside of the duplicated blocks. Within the identified duplicated blocks associated with the recent salicoid duplication event, 25% (4 of 16) of *Populus* neutral/alkaline invertase genes (*PtrNINV3*/*4*, *PtrNINV9*/*11*) were preferentially retained duplicates that located in both duplicated regions of two block pairs. *Populus* contains three pairs of paralogous neutral/alkaline invertase genes (*PtrNINV3*/*4*, *PtrNINV8*/*12*, *PtrNINV9*/*11*), based on chromosomal location and phylogenetic analyses (Fig 4 and S1 Fig). None of the *Populus* neutral/alkaline invertase genes was represented in distinct tandem duplicate gene clusters, which suggests that tandem duplications did not act significantly in the expansion of the neutral/alkaline invertase sub-family in *Populus*.

In this study, the Ka/Ks ratio of the three identified putative paralogous gene pairs identified was calculated to reveal the divergence fate after duplication of the *Populus* neutral/alkaline invertase genes. In addition, based on a divergence rate of 9.1 × 10^−9^ synonymous mutations per synonymous site per year proposed for *Populus* [21], the segmental duplications of the paralogous gene pairs in the *Populus* neutral/alkaline invertase sub-family were estimated to occur between 3.29 (Ks = 0.0598) to 13.49 (Ks = 0.2456) million years (MY) ago. The results of segmental duplications blocks showed that the Ka/Ks ratios of all paralogous pairs contained both > 1 and < 1 (Table 2).

**Table 2:** The Ka/Ks ratios and estimated divergence times for paralogous gene pairs.

Paralogous pairs	Identities (%)	Ka	Ks	Ka/Ks	Duplication date (MY)
*PtrNINV3*/*4*	90.92	0.0998	0.0598	1.6701	3.29
*PtrNINV8*/*12*	93.49	0.0279	0.2025	0.1378	11.13
*PtrNINV9*/*11*	91.01	0.0517	0.2456	0.2105	13.49

### Gene expression, invertase activity and sugar content in the tissues and organs of *P*. *tomentosa*

**Fig 6:**
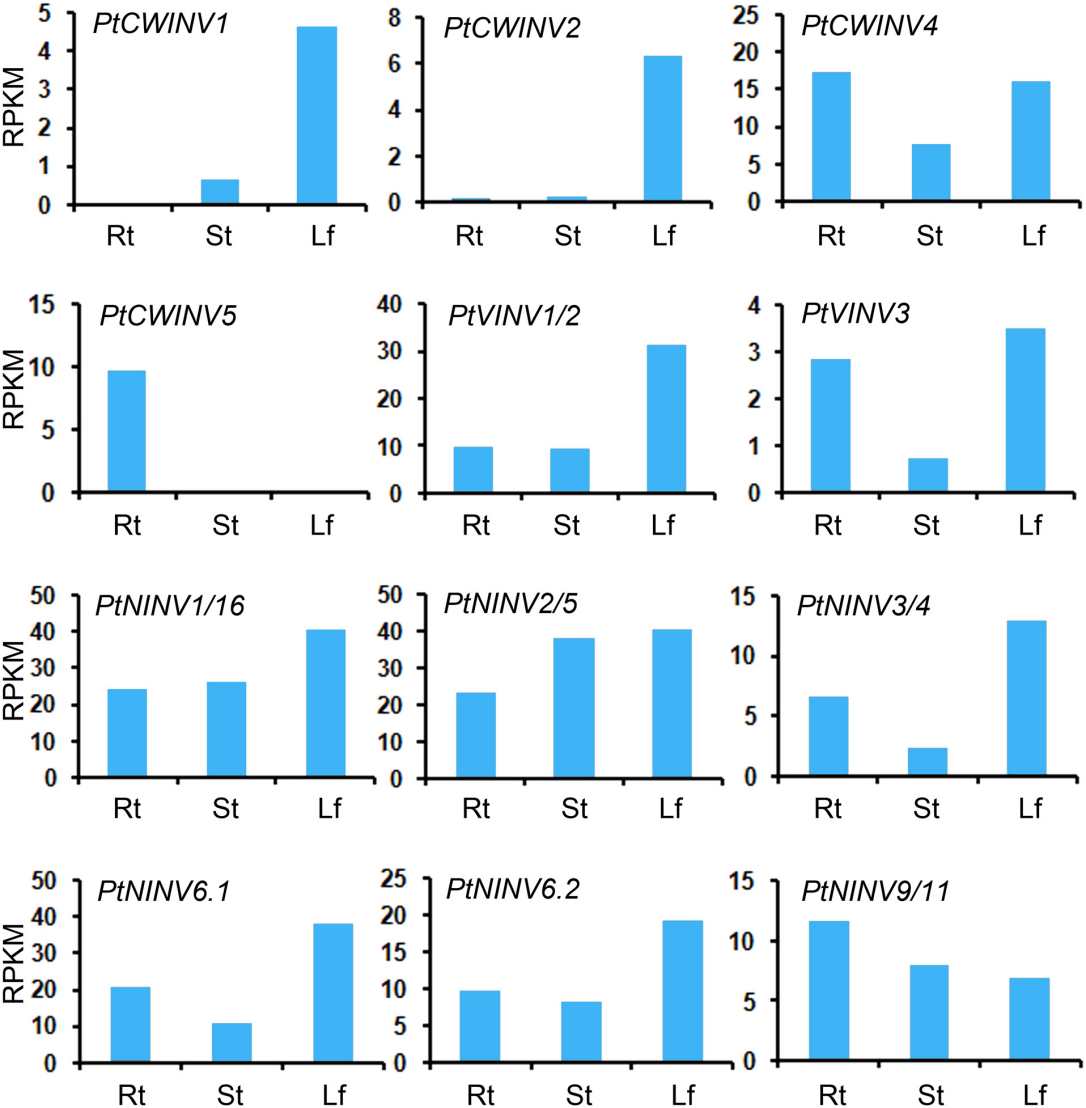
Expression analyses obtained by RNA-seq for *Populus* invertase genes in roots, stems and leaves. On the x-axis: Rt, roots; St, stems; Lf, leaves.

The expressions of *PtINV* family genes in roots, stems and leaves were examined using RNA-seq. *PtCWINV1* and *PtCWINV2* were strongly expressed in leaves, weakly expressed in stems, and almost not detected in roots. *PtCWINV5* was expressed only in roots (Fig 6). The expression patterns of *PtCWINV4*, *PtVINV1*/*2*, *PtVINV3*, *PtNINV3*/*4*, *PtNINV6*.*1*, and *PtNINV6*.*2* were similar, being constitutive and expressed at the lowest levels in stems. Similar expression patterns were also found for *PtNINV1*/*16*, and *PtNINV2*/*5*, with maximum expression in leaves and minimum in roots. The transcript levels of *PtNINV9*/*11* were highest in roots and lowest in leaves (Fig 6). The tissue-specific expression pattern of *PtINV*s provides a basis for understanding the function of invertases in poplar.

**Fig 7:**
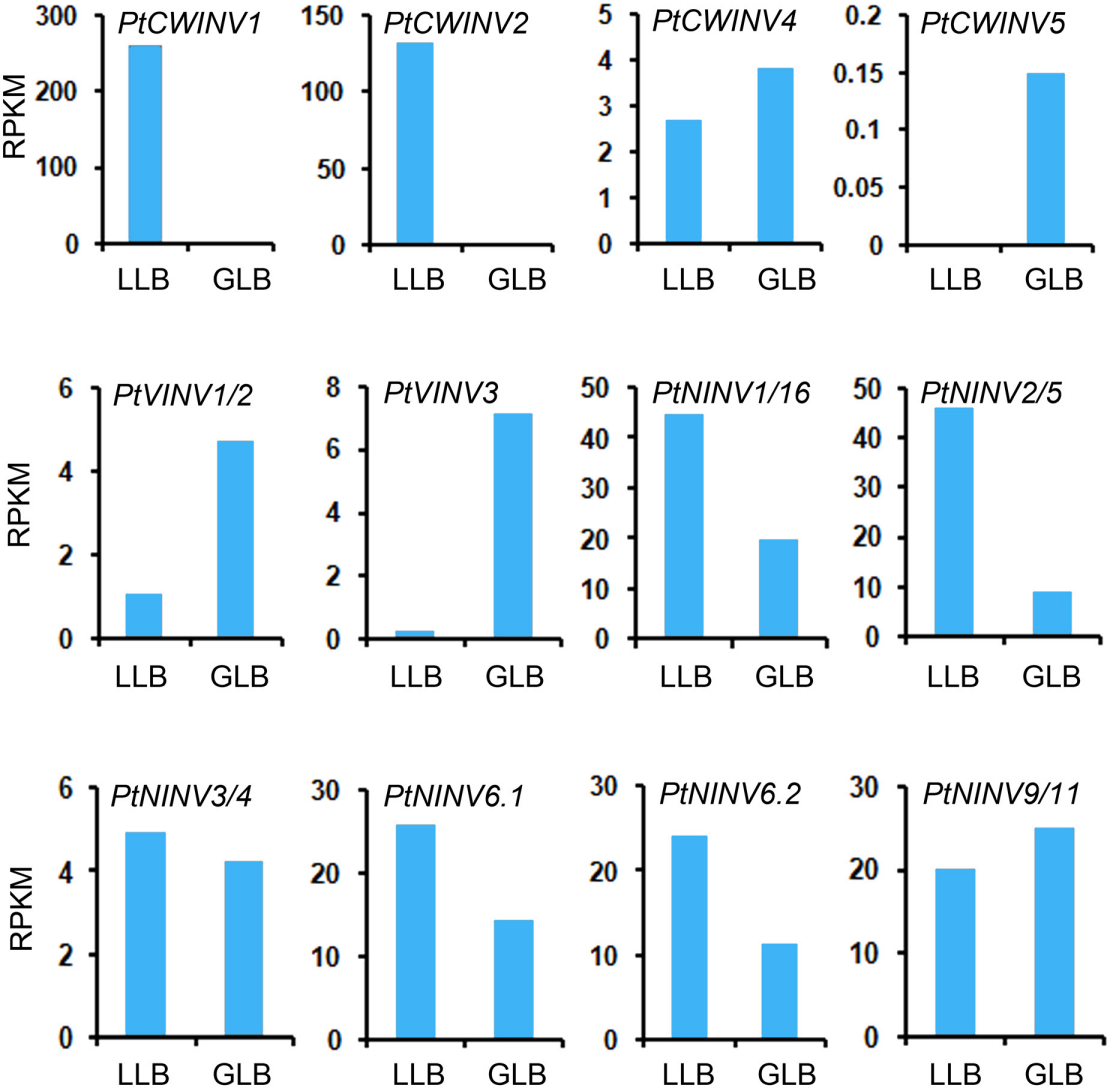
Expression profiles obtained by RNA-seq for *Populus* invertase genes in leaf buds. LLB, latent leaf buds; GLB, leaf buds at germination.

To investigate the role of invertase family genes in latent leaf buds and leaf buds during germination, the gene expression levels, invertase activities, and sugar contents were measured. The expression levels of *PtCWINV1* and *2*, *PtNINV1*/*16*, *2*/*5*, *3*/*4*, *6*.*1*, and *6*.*2* were higher in latent leaf buds, while those of *PtCWINV4* and *5*, *PtVINV1*/*2* and *3* and *PtNINV9*/*11* were lower in latent leaf buds (Fig 7).

**Fig 8:**
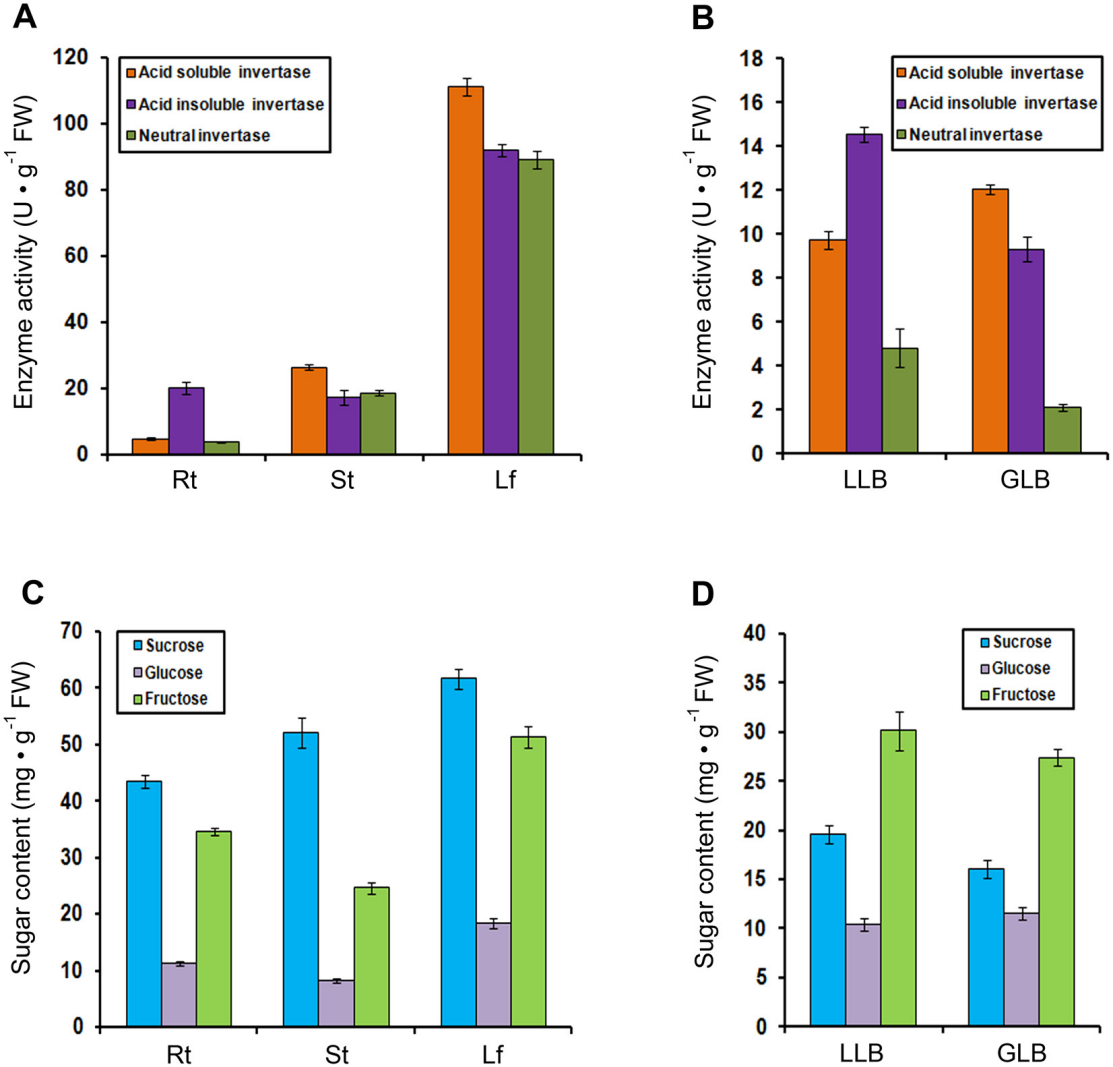
Enzyme activity profiles (acid-soluble, acid-insoluble and neutral invertases) in roots, stems, leaves (A), latent leaf buds and leaf buds at germination (B). Sugar content (sucrose, glucose and fructose) in roots, stems, leaves (C), latent leaf buds and leaf buds at germination (D). Rt, roots; St, stems; Lf, leaves; LLB, latent leaf buds; GLB, leaf buds at germination.

The activities of acid soluble and neutral invertases were highest in leaves, followed by stems and roots. The activity of acid insoluble invertase in leaves was considerably higher than in roots and stems (Fig 8A). Sucrose content was highest in leaves, followed by stems and roots. The patterns of glucose and fructose were similar, with the highest levels in leaves and the lowest in stems (Fig 8C). The activity of acid soluble invertase was increased and the activities of acid insoluble invertase and neutral invertase were decreased in leaf buds at germination (Fig 8B). A modest but detectable decrease in sucrose and fructose content occurred in leaf buds from dormancy to germination. Glucose content increased slightly in leaf buds at germination (Fig 8D).

### Invertase gene expression in response to abiotic and biotic stresses

**Fig 9:**
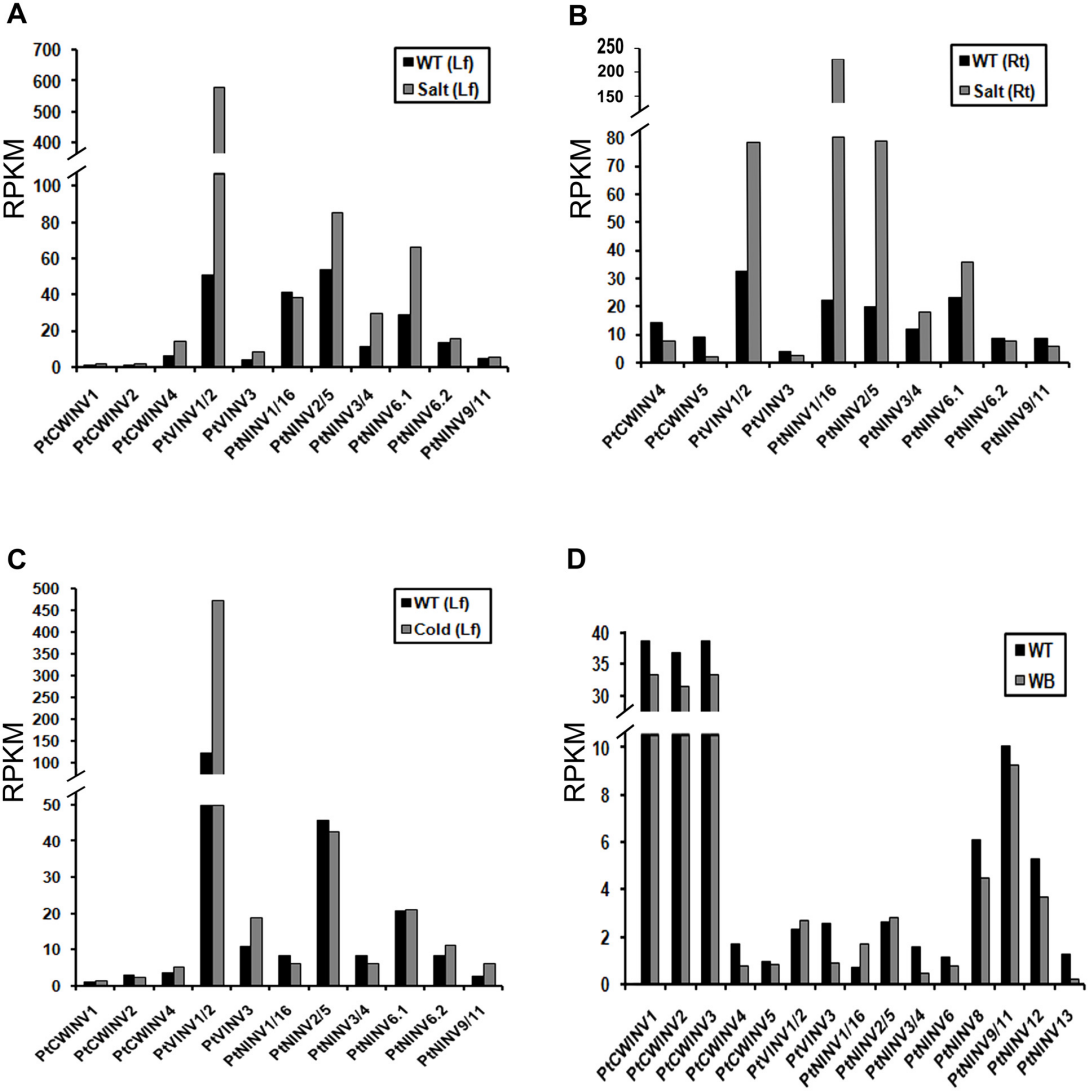
Expression profiles of *PtINV* gene members in response to salt/cold stress conditions (A-C) and pathogen infection (D). WT, wild type; WB, with *Botryosphaeria* infection; Lf, leaves; Rt, Roots.

To investigate the expression patterns of cell wall and vacuolar invertase genes in response to stress-related stimuli, the transcript levels of genes in leaves or roots under salt stress, in leaves under cold stress, and the response to infection by *Botryosphaeria dothidea* (which causes stem blister cankers) were analysed. In salt-treated leaves, four acid invertase genes (*PtCWINV4*, *PtVINV1*/*2*, and *PtVINV3*) were upregulated (Fig 9A). In salt-treated roots, two acid invertase genes (*PtCWINV4* and *5*) were downregulated, while *PtVINV1*/*2* were upregulated (Fig 9B). In leaves under cold stress, all vacuolar invertase genes were upregulated (Fig 9C). When infected by *B*. *dothidea*, five acid invertase genes (*PtCWINV1*, *2*, *3*, *4*, and *PtVINV3*) were downregulated (Fig 9D). In addition, the expression levels of neutral/alkaline invertase genes in response to stress-related stimuli were determined; the transcript levels of some genes were found to be substantially altered (Fig 9).

## Discussion

### Invertase gene family in *Populus*

A comparative genomic study revealed a ratio of 1.4–1.6 putative poplar homologs for each *Arabidopsis* gene [18]. In this study, the number of transcripts of invertase genes in *Populus* (45) is roughly 1.73-fold that of *Arabidopsis* (26). The greater abundance of invertase genes in the poplar genome is presumably due to the expansion of the gene families during genome duplication and the subsequent genomic evolution, and suggests a large range of functional roles for invertase genes in the more complex transcriptional regulation mechanisms of this woody species [31].

In the acid invertase sub-family, all eight genes contain the WECXDF motif (Fig 1 (7)); and six genes (exclude *PtrCWINV1* and *2*) contain the NDPNG motif (Fig 1 (2)). With the exception of *PtrVIN1*, the NDPNG motif is partly encoded by a mini-exon that contributes the tripeptide DPN to the second conserved motif of acid invertases, one of the smallest exons that are known by far in plants [32] (Fig 5A). Phylogenetic analysis revealed that the acid invertase sub-family could be separated into α and β clades (Fig 3). Several key features distinguish the α and β clades of the poplar acid invertases. The first two features are N- and C-terminal extensions, both of which are proposed to function in differential (cell wall and vacuole) targeting [2]. The third is the conserved WEC(P/V)DF motif containing a proline (P) in the α clade and a valine (V) in the β clade (Fig 1 (7)), which is one of the three requisite carboxylate groups for activity [33]. Although the significance of these differences is unclear, a previous study demonstrated that the (P/V) substitution modifies not only the pH optima but also substrate specificities of cell-wall and vacuolar invertases [34]. The NDPNG and WEC(P/V)DF motifs have been analysed in the rice invertase gene family [6,35]. A previous study hypothesised that *PtrVINV1* was formed later in the evolution of poplar as a processed transcript of *PtrVINV2* that was reinserted into the genome after reverse transcription [9]. To our knowledge, poplar vacuolar invertases constitute the first report of a plant with more than two vacuolar invertases, and *PtrVINV1* is the first reported intron-less invertase. Homologs of the three cell wall invertase genes (*PtrCWINV1*, *PtrCWINV2*, *PtrCWINV3*) were previously identified as cell wall invertase genes in *P*. *alba* × *grandidentata* (*Pa*×*gINV2*, *Pa*×*gINV3* and *Pa*×*gINV1*, respectively). The deduced amino acid sequences of *Pa*×*gINV*1, *Pa*×*gINV*2 and *Pa*×*gINV*3 also had conserved β-fructosidase (NDPNG) and cell-wall invertase (WEC(P/V)DF) motifs [36].

In the neutral/alkaline invertase sub-family, *PtrNINV13*-*16* are encoded by apparently incomplete ORFs [9]. They are missing significant portions of their coding regions. *PtrNINV13* is missing the first and last exons. *PtrNINV14* and *15* are missing the first and last exons and portions of the third exon. *PtrNINV16* contains only a short ORF encoding a portion of the third exon. A previous study showed that these four neutral/alkaline invertase genes retain conserved intron/exon splice sites, conserved motifs and the ability to be transcribed. The transcriptional evidence shows that these genes are not “pseudogenes”, which are transcriptionally and translationally silent [37]. However, in eukaryotic organisms, there are examples of transcribed pseudogenes, such as in *Arabidopsis* and liverworts [38]. The pseudogenes could function as negative regulators of the transcription of their ancestral gene in a manner similar to the *cis*-elements in the promoter of pseudogene derived from human cytokeratin 17, which can interact with distal elements in the promoter of the functional gene to regulate transcriptional activity [39,40]. Thus, we hypothesise that *PtrNINV13*-*16* may have biologically relevant functions in gene regulation, in contrast to invertase enzyme activity.

### The genome duplication events of *INV* genes in *Populus*

A previous study revealed that an ancient eurosid genome duplication event produced vacuolar invertases *PtrVINV2* and *PtrVINV3*. A tandem duplication produced the *PtrCWINV1*/*2* and *PtrCWINV4*/*5* pairs and probably took place after the poplar and *Arabidopsis* speciation event [9]. Previous studies revealed that the *Populus* genome has undergone at least three rounds of genome-wide duplication followed by multiple segmental duplication, tandem duplication and transposition events, such as retroposition and replicative transpositions [18,41]. The segmental duplication concerning the salicoid duplication event that occurred 65 million years (MY) ago significantly promoted to the expansion of many multi-gene families [31,42–45]. Based on the location of pairs of paralogous genes on different chromosomes and the genomic organisation of the *Populus* neutral/alkaline invertase genes, we conclude that segmental duplications may have contributed to the expansion of the *Populus* neutral/alkaline invertase sub-family, but the effect of segmental duplication was not so significant as for other multi-gene families in *Populus* [31,42–45]. In addition, our results indicate that *Populus* neutral/alkaline invertase genes were preferentially retained at a relatively high rate of 25%, which is lower than the average rate (33%) following the salicoid genome-wide duplication in the *Populus* lineage [18], suggesting that only segmental duplication is involved in the expansion of the *Populus* neutral/alkaline invertase sub-family. In contrast, high retention rates for duplicated genes were observed in some other *Populus* gene families [42,44,46].

The substitution rate ratio of nonsynonymous (dN or Ka) versus synonymous (dS or Ks) is an indicator of selection history on genes or gene regions. The approximate dates of duplication events were calculated using Ks. Generally, Ka/Ks < 1 indicates a functional constraint with negative or purifying selection of the genes, Ka/Ks > 1 indicates accelerated evolution with positive selection and Ka/Ks = 1 suggests neutral selection [47]. As the results of segmental duplications blocks showed that the Ka/Ks ratios of all paralogous pairs were both > 1 and < 1 (Table 2), we conclude that the *Populus* neutral/alkaline invertase sub-family has undergone both positive and negative selection pressures with limited functional divergence after segmental duplication. In grapes, estimation of Ks showed the ancient origin of all neutral invertase genes and the lack of expansion by gene duplication past the event of polyploidisation [11].

### Differential expression patterns of multiple members of *INV* gene family in poplar

Most *PtINV* genes were expressed in all roots, stems and leaves, but with organ-specific regulation. In the sugar beet, the extracellular invertase gene *BVInv*-*CW1* was almost exclusively expressed in roots and may be involved in the regulation of sink strength in sucrose-storing tap roots during the early stages of development [48]. Our results indicated that most *PtINV* genes were highly expressed in leaves. This was also the case in the neutral invertase gene family of grapes and some alkaline/neutral invertase genes of cassava [11,49]. The differential expression patterns of *INV*s in various tissues reveals that these genes play an important role in their respective organs in terms of providing carbohydrates for growth and development [49,50]. In carrots and tomatoes, some cell wall or vacuolar invertase genes showed markedly different organ- and development-stage-specific expression patterns, and both contain a flower-specific acid invertase gene [51–53]. These findings suggested that plants have evolved a small acid invertase gene family that is expressed independently at specific times and tissues during development [2]. In this study, both sucrose content and invertase activity were highest in leaves (source organs). There are three possible reasons for this phenomenon. First, the leaf is primarily involved in photosynthesis, and sucrose is one of the major end products of this process. Second, invertase is just one of many enzymes (e.g. SS and SPS) that play important roles in sugar metabolism. Third, this perhaps suggests that lower efficiency of sucrose hydrolysis by invertase exists in source organs. Low efficiency of sucrose hydrolysis was also reported in previous studies of cassava [49] and grape berries [54]. In the present study, the activity of acid soluble invertase was increased in leaf buds at germination and the activities of acid insoluble invertase and neutral invertase were decreased in latent leaf buds. Similar results were found for enzyme activities and gene expression levels in sucrose metabolism in relation to sugar accumulation and composition in the aril of *Litchi chinensis* Sonn. [26]. Traditional extraction and purification procedures are often complicated and sometimes lead to low yield. Additional complication (multiple isoforms) frequently arises when attempting to purify plant enzymes from native tissue. In a previous study by Canam et al. [55], to avoid the difficulty in purifying distinct invertases to complete homogeneity from native tissues, the *Pichia pastoris* expression system was used to heterologously express and characterize two hybrid poplar cell wall invertases (Pa×gINV1 and Pa×gINV2). The results showed that these two enzymes had distinct pH optima and temperature optima, as well as the ability to hydrolyze the fructose from sucrose and other fructofuranosides such as raffinose, stachyose and verbascose, with PaxgINV2 having higher affinity for each of the substrates tested.

A previous study by Canam et al. [36] showed that the expression of *Pa*×*gINV1* was associated with dormancy, while *Pa*×*gINV2* expression was prominent in tissues undergoing active growth and expansion, the phloem and buds coinciding with bud break, the emerging shoots, as well as the apical, petiole and leaf tissues of newly formed branch. In the present study, *PtCWINV1* (the homolog of *Pa*×*gINV2*) was mainly expressed in leaf and latent leaf buds (Figs 6 and 7). Previous study concluded that *Pa*×*gINV3* was a floral-specific gene because no *Pa*×*gINV3* transcripts were detected in any of the tissues examined (the expression in the reproductive tissue was not investigated) in *P*. *alba* × *grandidentata* [36] and the homolog to *Pa*×*gINV3* (*PtCIN2*) was weakly expressed solely in the catkin of field-grown *P*. *deltoides* [9]. In our study, *PtCWINV2* (the homolog of *Pa*×*gINV3*) was expressed in later stages of developing male and female floral buds (unpublished data), but it was also expressed in some other tissues, such as leaf and latent leaf buds (Figs 6 and 7).

### Genes for invertases are regulated by salt/cold stress and pathogens

Previous studies indicated that acid invertase genes can be regulated by sugars [51] or by several stress factors, including pathogens, wounding, water stress, and cold [2,7]. In this study, in salt-treated leaves, *PtCWINV4*, *PtVINV1*/*2*, and *PtVINV3* were upregulated. In salt-treated roots, *PtCWINV4* and *5* were downregulated, while *PtVINV1*/*2* were upregulated. In leaves under cold stress, all vacuolar invertase genes were upregulated. In tulip (*Tulipa gesneriana* L. cv. Apeldoorn) bulbs invertase mRNA levels were substantially upregulated as a result of cold stress [56]. Previous studies have reported a correlation between increased acid invertase activity and infection of plants by various pathogens [7]. In our study, *PtCWINV1*, *2*, *3*, *4*, and *PtVINV3* were downregulated when infected by *B*. *dothidea*. Nevertheless, in carrot tap roots, the response to infection by *Erwinia carotovora*, a bacterial pathogen, was extremely rapid and transient [7]. The expressions peaked 1 h after first contact with the pathogen and decreased rapidly thereafter. Moreover, induction of gene expression by pathogen infection seems not to be systemic, but is dependent on the infection site [57].

## Supporting Information

S1 Fig Physical locations of invertase genes on *Populus* chromosomes. Chromosome numbers and sizes (Mb) are indicated at the bottom of each chromosome. Chromosomal positions of the poplar invertase genes are indicated by gene names. The lines connect corresponding pairs of paralogous genes in both duplicated blocks. The scale bar represents a 2.0-Mb chromosomal distance.
(TIF)

S1 Table Characteristics of acid invertase sub-family members in *Populus trichocarpa*. (DOCX)

S2 Table Characteristics of neutral/alkaline invertase sub-family members in *Populus trichocarpa*. (DOCX)

S3 Table Coding region nucleotide (upper portion of matrix) and amino acid (bottom portion of matrix) sequence pairwise comparison (% identity) between poplar acid invertase sub-family genes. (DOCX)

S4 Table Coding region nucleotide (upper portion of matrix) and amino acid (bottom portion of matrix) sequence pairwise comparison (% identity) between poplar neutral/alkaline invertase sub-family genes. (DOCX)

## Abbreviations

CWINV: cell wall invertase
INV: invertase
NINV: neutral/ alkaline invertase
SPS: sucrose phosphate synthase
SS: sucrose synthase
VINV: vacuolar invertase

## References

[1] Roitsch T, Gonzalez MC (2004) Function and regulation of plant invertases: sweet sensations. Trends in Plant Science 9: 606–613. 15564128 10.1016/j.tplants.2004.10.009

[2] Sturm A, Tang GQ (1999) The sucrose-cleaving enzymes of plants are crucial for development, growth and carbon partitioning. Trends in Plant Science 4: 401–407. 10498964 10.1016/s1360-1385(99)01470-3

[3] Sturm A (1999) Invertases. Primary structures, functions, and roles in plant development and sucrose partitioning. Plant Physiology 121: 1–8. 10482654 10.1104/pp.121.1.1 PMC1539224

[4] Haouazine-Takvorian N, Tymowska-Lalanne Z, Takvorian A, Tregear J, Lejeune B, Lecharny A, et al (1997) Characterization of two members of the *Arabidopsis thaliana* gene family, *At beta fruct3* and *At beta fruct4*, coding for vacuolar invertases. Gene 197: 239–251. 9332372 10.1016/s0378-1119(97)00268-0

[5] Sherson SM, Alford HL, Forbes SM, Wallace G, Smith SM (2003) Roles of cell-wall invertases and monosaccharide transporters in the growth and development of *Arabidopsis* . Journal of Experimental Botany 54: 525–531. 12508063 10.1093/jxb/erg055

[6] Ji X, Van den Ende W, Van Laere A, Cheng S, Bennett J (2005) Structure, evolution, and expression of the two invertase gene families of rice. Journal of Molecular Evolution 60: 615–634. 15983871 10.1007/s00239-004-0242-1

[7] Sturm A, Chrispeels MJ (1990) cDNA cloning of carrot extracellular beta-fructosidase and its expression in response to wounding and bacterial infection. Plant Cell 2: 1107–1119. 2152110 10.1105/tpc.2.11.1107 PMC159958

[8] Vargas W, Cumino A, Salerno GL (2003) Cyanobacterial alkaline/neutral invertases. Origin of sucrose hydrolysis in the plant cytosol? Planta 216: 951–960. 12687362 10.1007/s00425-002-0943-x

[9] Bocock PN, Morse AM, Dervinis C, Davis JM (2008) Evolution and diversity of invertase genes in *Populus trichocarpa* . Planta 227: 565–576. 17938954 10.1007/s00425-007-0639-3

[10] Welham T, Pike J, Horst I, Flemetakis E, Katinakis P, Kaneko T, et al (2009) A cytosolic invertase is required for normal growth and cell development in the model legume, *Lotus japonicus* . Journal of Experimental Botany 60: 3353–3365. 10.1093/jxb/erp169 19474088 PMC2724688

[11] Nonis A, Ruperti B, Pierasco A, Canaguier A, Adam-Blondon AF, Di Gaspero G, et al (2008) Neutral invertases in grapevine and comparative analysis with *Arabidopsis*, poplar and rice. Planta 229: 129–142. 10.1007/s00425-008-0815-0 18800225

[12] Rossouw D, Kossmann J, Botha FC, Groenewald J-H (2010) Reduced neutral invertase activity in the culm tissues of transgenic sugarcane plants results in a decrease in respiration and sucrose cycling and an increase in the sucrose to hexose ratio. Functional Plant Biology 37: 22–31.

[13] Nonis A, Ruperti B, Falchi R, Casatta E, Thamasebi Enferadi S, Vizzotto G (2007) Differential expression and regulation of a neutral invertase encoding gene from peach (*Prunus persica*): evidence for a role in fruit development. Physiologia Plantarum 129: 436–446.

[14] Swarbreck D, Wilks C, Lamesch P, Berardini TZ, Garcia-Hernandez M, Foerster H, et al (2008) The *Arabidopsis* Information Resource (TAIR): gene structure and function annotation. Nucleic Acids Research 36: D1009–1014. 17986450 10.1093/nar/gkm965 PMC2238962

[15] Goodstein DM, Shu S, Howson R, Neupane R, Hayes RD, Fazo J, et al (2012) Phytozome: a comparative platform for green plant genomics. Nucleic Acids Research 40: D1178–1186. 10.1093/nar/gkr944 22110026 PMC3245001

[16] Guo AY, Zhu QH, Chen X, Luo JC (2007) GSDS: a gene structure display server. Yi Chuan 29: 1023–1026. 17681935

[17] Tamura K, Stecher G, Peterson D, Filipski A, Kumar S (2013) MEGA6: Molecular Evolutionary Genetics Analysis version 6.0. Molecular Biology and Evolution 30: 2725–2729. 10.1093/molbev/mst197 24132122 PMC3840312

[18] Tuskan GA, Difazio S, Jansson S, Bohlmann J, Grigoriev I, Hellsten U, et al (2006) The genome of black cottonwood, *Populus trichocarpa* (Torr. & Gray). Science 313: 1596–1604. 16973872 10.1126/science.1128691

[19] Ouyang S, Zhu W, Hamilton J, Lin H, Campbell M, Childs K, et al (2007) The TIGR Rice Genome Annotation Resource: improvements and new features. Nucleic Acids Research 35: D883–887. 17145706 10.1093/nar/gkl976 PMC1751532

[20] Yang Z (2007) PAML 4: phylogenetic analysis by maximum likelihood. Molecular Biology and Evolution 24: 1586–1591. 17483113 10.1093/molbev/msm088

[21] Lynch M, Conery JS (2000) The evolutionary fate and consequences of duplicate genes. Science 290: 1151–1155. 11073452 10.1126/science.290.5494.1151

[22] Ottow EA, Brinker M, Teichmann T, Fritz E, Kaiser W, Brosche M, et al (2005) *Populus euphratica* displays apoplastic sodium accumulation, osmotic adjustment by decreases in calcium and soluble carbohydrates, and develops leaf succulence under salt stress. Plant Physiology 139: 1762–1772. 16299175 10.1104/pp.105.069971 PMC1310557

[23] Liao W, Ji L, Wang J, Chen Z, Ye M, Ma H, et al (2014) Identification of glutathione S-transferase genes responding to pathogen infestation in *Populus tomentosa* . Functional & Integrative Genomics 14: 517–529. 24870810 10.1007/s10142-014-0379-y

[24] Chen Z, Wang J, Ye MX, Li H, Ji LX, Li Y, et al (2013) A Novel Moderate Constitutive Promoter Derived from Poplar (*Populus tomentosa* Carrière). International Journal of Molecular Sciences 14: 6187–6204. 10.3390/ijms14036187 23507754 PMC3634493

[25] Vizzotto G, Pinton R, Varanini Z, Costa G (1996) Sucrose accumulation in developing peach fruit. Physiologia Plantarum 96: 225–230.

[26] Yang Z, Wang T, Wang H, Huang X, Qin Y, Hu G (2013) Patterns of enzyme activities and gene expressions in sucrose metabolism in relation to sugar accumulation and composition in the aril of *Litchi chinensis* Sonn. Journal of Plant Physiology 170: 731–740. 10.1016/j.jplph.2012.12.021 23499454

[27] Li M, Feng F, Cheng L (2012) Expression patterns of genes involved in sugar metabolism and accumulation during apple fruit development. PLoS ONE 7: e33055 10.1371/journal.pone.0033055 22412983 PMC3296772

[28] Zhang HP, Wu JY, Qin GH, Yao GF, Qi KJ, Wang LF, et al (2014) The role of sucrose-metabolizing enzymes in pear fruit that differ in sucrose accumulation. Acta Physiologiae Plantarum 36: 71–77.

[29] Ye M, Chen Z, Su X, Ji L, Wang J, Liao W, et al (2014) Study of seed hair growth in *Populus tomentosa*, an important character of female floral bud development. BMC Genomics 15: 475 10.1186/1471-2164-15-475 24929561 PMC4089023

[30] Mortazavi A, Williams BA, McCue K, Schaeffer L, Wold B (2008) Mapping and quantifying mammalian transcriptomes by RNA-Seq. Nature Methods 5: 621–628. 10.1038/nmeth.1226 18516045 PMC13303166

[31] Hu R, Chi X, Chai G, Kong Y, He G, Wang X, et al (2012) Genome-wide identification, evolutionary expansion, and expression profile of homeodomain-leucine zipper gene family in poplar (*Populus trichocarpa*). PLoS ONE 7: e31149 10.1371/journal.pone.0031149 22359569 PMC3281058

[32] Bournay AS, Hedley PE, Maddison A, Waugh R, Machray GC (1996) Exon skipping induced by cold stress in a potato invertase gene transcript. Nucleic Acids Research 24: 2347–2351. 8710506 10.1093/nar/24.12.2347 PMC145944

[33] Alberto F, Bignon C, Sulzenbacher G, Henrissat B, Czjzek M (2004) The three-dimensional structure of invertase (beta-fructosidase) from *Thermotoga maritima* reveals a bimodular arrangement and an evolutionary relationship between retaining and inverting glycosidases. Journal of Biological Chemistry 279: 18903–18910. 14973124 10.1074/jbc.M313911200

[34] Goetz M, Roitsch T (1999) The different pH optima and substrate specificities of extracellular and vacuolar invertases from plants are determined by a single amino-acid substitution. Plant Journal 20: 707–711. 10652142 10.1046/j.1365-313x.1999.00628.x

[35] Cho JI, Lee SK, Ko S, Kim HK, Jun SH, Lee YH, et al (2005) Molecular cloning and expression analysis of the cell-wall invertase gene family in rice (*Oryza sativa* L.). Plant Cell Reports 24: 225–236. 15759120 10.1007/s00299-004-0910-z

[36] Canam T, Mak SW, Mansfield SD (2008) Spatial and temporal expression profiling of cell-wall invertase genes during early development in hybrid poplar. Tree Physiology 28: 1059–1067. 18450570 10.1093/treephys/28.7.1059

[37] Li W-H, Gojobori T, Nei M (1981) Pseudogenes as a paradigm of neutral evolution. Nature 292: 237–239. 7254315 10.1038/292237a0

[38] Balakirev ES, Chechetkin VR, Lobzin VV, Ayala FJ (2003) DNA polymorphism in the beta-Esterase gene cluster of *Drosophila melanogaster* . Genetics 164: 533–544. 12807774 10.1093/genetics/164.2.533 PMC1462603

[39] McCarrey JR, Riggs AD (1986) Determinator-inhibitor pairs as a mechanism for threshold setting in development: a possible function for pseudogenes. Proceedings of the National Academy of Sciences of the United States of America 83: 679–683. 2418440 10.1073/pnas.83.3.679 PMC322927

[40] Troyanovsky SM, Leube RE (1994) Activation of the silent human cytokeratin 17 pseudogene-promoter region by cryptic enhancer elements of the cytokeratin 17 gene. European Journal of Biochemistry 225: 61–69. 7523124 10.1111/j.1432-1033.1994.00061.x

[41] Brunner AM, Busov VB, Strauss SH (2004) Poplar genome sequence: functional genomics in an ecologically dominant plant species. Trends in Plant Science 9: 49–56. 14729219 10.1016/j.tplants.2003.11.006

[42] Barakat A, Bagniewska-Zadworna A, Choi A, Plakkat U, DiLoreto DS, Yellanki P, et al (2009) The cinnamyl alcohol dehydrogenase gene family in *Populus*: phylogeny, organization, and expression. BMC Plant Biology 9: 26 10.1186/1471-2229-9-26 19267902 PMC2662859

[43] Barakat A, Choi A, Yassin NB, Park JS, Sun Z, Carlson JE (2011) Comparative genomics and evolutionary analyses of the O-methyltransferase gene family in *Populus* . Gene 479: 37–46. 10.1016/j.gene.2011.02.008 21338660

[44] Kalluri UC, Difazio SP, Brunner AM, Tuskan GA (2007) Genome-wide analysis of *Aux*/*IAA* and *ARF* gene families in *Populus trichocarpa* . BMC Plant Biology 7: 59 17986329 10.1186/1471-2229-7-59 PMC2174922

[45] Zuo R, Hu R, Chai G, Xu M, Qi G, Kong Y, et al (2013) Genome-wide identification, classification, and expression analysis of CDPK and its closely related gene families in poplar (*Populus trichocarpa*). Molecular Biology Reports 40: 2645–2662. 10.1007/s11033-012-2351-z 23242656

[46] Tuominen LK, Johnson VE, Tsai CJ (2011) Differential phylogenetic expansions in BAHD acyltransferases across five angiosperm taxa and evidence of divergent expression among *Populus* paralogues. BMC Genomics 12: 236 10.1186/1471-2164-12-236 21569431 PMC3123328

[47] Yang X, Tuskan GA, Cheng MZ (2006) Divergence of the *Dof* gene families in poplar, *Arabidopsis*, and rice suggests multiple modes of gene evolution after duplication. Plant Physiology 142: 820–830. 16980566 10.1104/pp.106.083642 PMC1630746

[48] Gonzalez MC, Roitsch T, Cejudo FJ (2005) Circadian and developmental regulation of vacuolar invertase expression in petioles of sugar beet plants. Planta 222: 386–395. 16052318 10.1007/s00425-005-1542-4

[49] Yao Y, Geng M-T, Wu X-H, Liu J, Li R-M, Hu X-W, et al (2014) Genome-Wide Identification, Expression, and Activity Analysis of Alkaline/Neutral Invertase Gene Family from Cassava (*Manihot esculenta* Crantz). Plant Molecular Biology Reporter 33: 304–315. 10.3390/ijms15057313 PMC4057674 24786092

[50] Wang Y, Chen J, Feng J, Qin Q, Huang J (2015) Overexpression of a loquat (*Eriobotrya japonica* Lindl.) vacuolar invertase affects sucrose levels and growth. Plant Cell, Tissue and Organ Culture 10.1007/s11240-015-0817-0

[51] Godt DE, Roitsch T (1997) Regulation and tissue-specific distribution of mRNAs for three extracellular invertase isoenzymes of tomato suggests an important function in establishing and maintaining sink metabolism. Plant Physiology 115: 273–282. 9306701 10.1104/pp.115.1.273 PMC158483

[52] Lorenz K, Lienhard S, Sturm A (1995) Structural organization and differential expression of carrot beta-fructofuranosidase genes: identification of a gene coding for a flower bud-specific isozyme. Plant Molecular Biology 28: 189–194. 7787183 10.1007/BF00042049

[53] Sturm A, Šebková V, Lorenz K, Hardegger M, Lienhard S, Unger C (1995) Development-and organ-specific expression of the genes for sucrose synthase and three isoenzymes of acid β-fructofuranosidase in carrot. Planta 195: 601–610.

[54] Xie Z, Li B, Forney CF, Xu W, Wang S (2009) Changes in sugar content and relative enzyme activity in grape berry in response to root restriction. Scientia horticulturae 123: 39–45.

[55] Canam T, Unda F, Mansfield SD (2008) Heterologous expression and functional characterization of two hybrid poplar cell-wall invertases. Planta 228: 1011–1019. 10.1007/s00425-008-0801-6 18704491

[56] Balk PA, de Boer AD (1999) Rapid stalk elongation in tulip (*Tulipa gesneriana* L. cv. Apeldoorn) and the combined action of cold-induced invertase and the water-channel protein gammaTIP. Planta 209: 346–354. 10502102 10.1007/s004250050642

[57] Benhamou N, Grenier J, Chrispeels MJ (1991) Accumulation of beta-fructosidase in the cell walls of tomato roots following infection by a fungal wilt pathogen. Plant Physiology 97: 739–750. 16668461 10.1104/pp.97.2.739 PMC1081069

