# Supplementary figures and images for: Genome-Wide Identification of the Invertase Gene Family in Populus
Source: PLoS One. 2015 Sep 22;10(9):e0138540. doi: 10.1371/journal.pone.0138540 (PMC4579127; doi:10.1371/journal.pone.0138540)

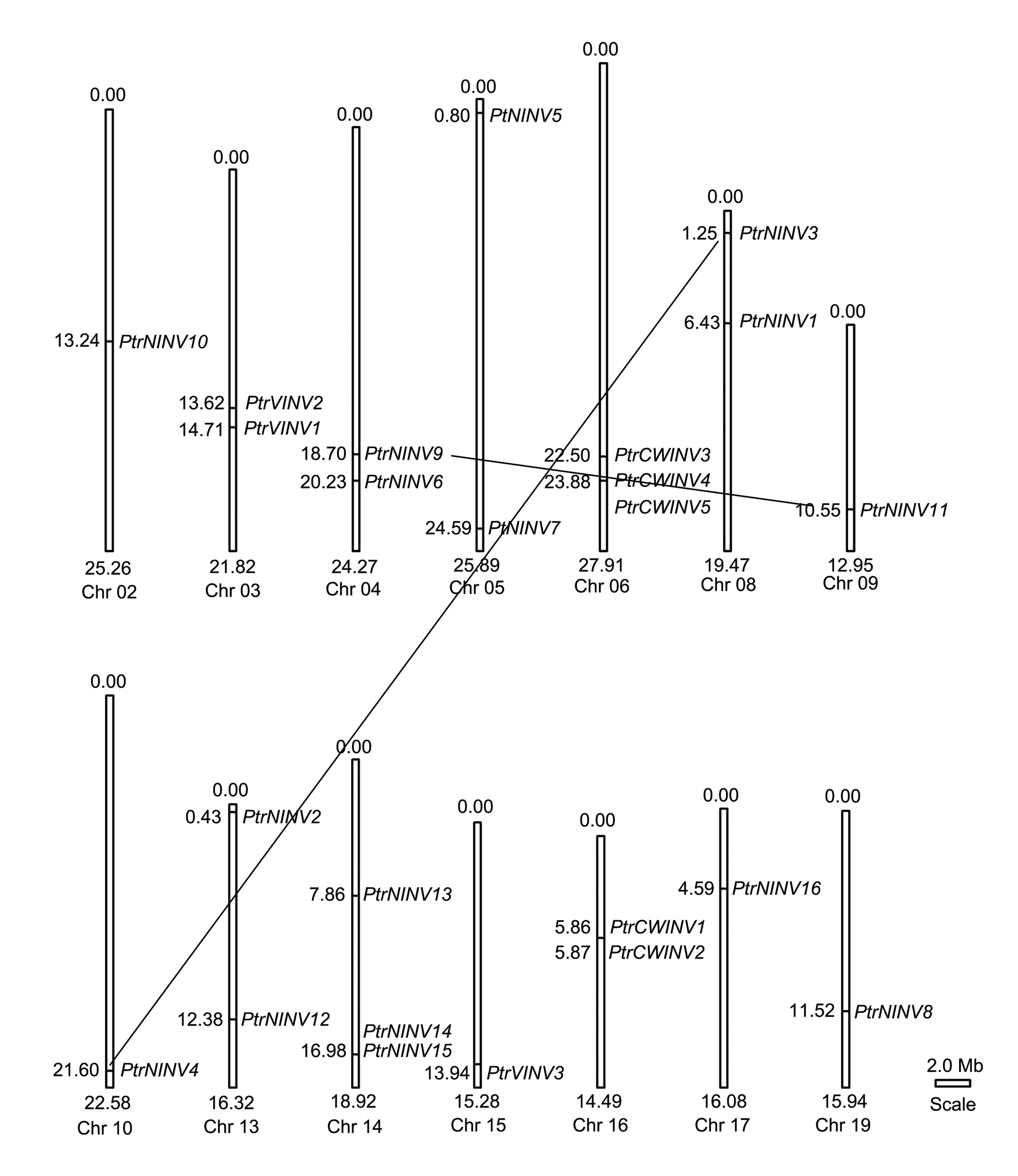

Supplement: S1 Fig — Chromosome numbers and sizes (Mb) are indicated at the bottom of each chromosome. Chromosomal positions of the poplar invertase genes are indicated by gene names. The lines connect corresponding pairs of paralogous genes in both duplicated blocks. The scale bar represents a 2.0-Mb chromosomal distance. (TIF) [file pone.0138540.s001.tif]
