# Supplementary material for: Genome-Wide Identification of the Invertase Gene Family in Populus
Source: PLoS One. 2015 Sep 22;10(9):e0138540. doi: 10.1371/journal.pone.0138540 (PMC4579127; doi:10.1371/journal.pone.0138540)
Supplement: S1 Table — (DOCX) [file pone.0138540.s002.docx]

**S1 Table. Characteristics of acid invertase sub-family members in *Populus trichocarpa*.**

| **Gene name** | **Predicted compartment** | **Transcript name** | **gDNA size (nts)** | **Transcript size (nts)** | **CDS size (nts)** | **Protein size** | | **pI** | **Functional domains (start-end)** | |
| --- | --- | --- | --- | --- | --- | --- | --- | --- | --- | --- |
|  |  |  |  |  |  | **Peptide residues** | **Mw (kDa)** |  | ***a* (N)** | ***b* (C)** |
| **Acid invertases** | |  |  |  |  |  |  |  |  |  |
| *PtrCWINV1* | Cell wall | Potri.016G077400.1 | 3897 | 1947 | 1740 | 579 | 65.44 | 7 | 52-367 | 444-528 |
|  |  | Potri.016G077400.2 | 3897 | 1941 | 1734 | 576 | 65.13 | 6.79 | 52-367 | 444-526 |
| *PtrCWINV2* | Cell wall | Potri.016G077500.1 | 3799 | 1841 | 1701 | 566 | 63.32 | 6.51 | 60-347 | 424-506 |
| *PtrCWINV3* | Cell wall | Potri.006G210600.1 | 4711 | 1905 | 1737 | 578 | 65.5 | 8.63 | 51-368 | 444-527 |
| *PtrCWINV4* | Cell wall | Potri.006G227500.1 | 3599 | 2548 | 1731 | 576 | 65.6 | 5.03 | 51-367 | 441-524 |
|  |  | Potri.006G227500.2 | 3599 | 2544 | 1254 | 417 | 48.03 | 5.28 | 51-367 |  |
|  |  | Potri.006G227500.3 | 3434 | 2561 | 1506 | 501 | 57.21 | 5.16 | 51-367 | 441-490 |
| *PtrCWINV5* | Cell wall | Potri.006G227400.1 | 2756 | 1713 | 1713 | 570 | 64.77 | 8.03 | 42-357 | 425-514 |
| *PtrVINV1* | Vacuole | Potri.003G126300.1 | 1605 | 1605 | 1605 | 534 | 59.78 | 5.44 | 10-327 | 393-482 |
| *PtrVINV2* | Vacuole | Potri.003G112600.1 | 5303 | 2655 | 1989 | 662 | 73.33 | 5.81 | 136-454 | 525-609 |
|  |  | Potri.003G112600.2 | 5303 | 2659 | 1485 | 494 | 54.9 | 6.17 | 136-454 |  |
|  |  | Potri.003G112600.3 | 5303 | 2651 | 1413 | 470 | 52.08 | 5.37 | 1-262 | 333-417 |
|  |  | Potri.003G112600.4 | 5303 | 2763 | 1797 | 598 | 66.16 | 5.6 | 136-454 | 525-597 |
|  |  | Potri.003G112600.5 | 5303 | 2870 | 1704 | 567 | 62.77 | 5.58 | 136-454 |  |
|  |  | Potri.003G112600.6 | 5303 | 2739 | 1488 | 495 | 54.9 | 5.92 | 136-454 |  |
|  |  | Potri.003G112600.7 | 5303 | 2762 | 1704 | 567 | 62.77 | 5.58 | 136-454 |  |
|  |  | Potri.003G112600.8 | 3571 | 2293 | 1413 | 470 | 52.08 | 5.37 | 1-262 | 333-417 |
| *PtrVINV3* | Vacuole | Potri.015G127100.1 | 4673 | 2228 | 1920 | 639 | 71.77 | 4.98 | 118-437 | 499-586 |
|  |  | Potri.015G127100.2 | 4534 | 2080 | 1587 | 528 | 59.61 | 4.92 | 10-326 | 388-475 |
|  |  | Potri.015G127100.3 | 2764 | 1867 | 1569 | 522 | 58.63 | 4.91 | 7-320 | 382-469 |

***a***: Glycosyl hydrolases family 32 N-terminal domain; ***b***: Glycosyl hydrolases family 32 C terminal.
