# Supplementary material for: Genome-Wide Identification of the Invertase Gene Family in Populus
Source: PLoS One. 2015 Sep 22;10(9):e0138540. doi: 10.1371/journal.pone.0138540 (PMC4579127; doi:10.1371/journal.pone.0138540)
Supplement: S2 Table — (DOCX) [file pone.0138540.s003.docx]

**S2 Table. Characteristics of neutral/alkaline invertase sub-family members in *Populus trichocarpa*.**

| **Gene name** | **Predicted compartment** | **Transcript name** | **gDNA size(nts)** | **Transcript size (nts)** | **CDS size (nts)** | **Protein size** | | **pI** | **Functional domains (start-end)** | | |
| --- | --- | --- | --- | --- | --- | --- | --- | --- | --- | --- | --- |
|  |  |  |  |  |  | **Peptide residues** | **Mw (kDa)** |  | **Plant neutral invertase** | | |
| **Neutral /alkaline invertases** | |  |  |  |  |  |  |  |  |  |  |
| *PtrNINV1* | Cytosol | Potri.008G101500.1 | 4260 | 2757 | 2016 | 671 | 75.73 | 6.02 | 181-648 | | |
|  |  | Potri.008G101500.2 | 4260 | 2820 | 2079 | 692 | 78.12 | 6.03 | 202-669 | | |
|  |  | Potri.008G101500.3 | 4134 | 2567 | 2079 | 692 | 78.12 | 6.03 | 202-669 | | |
| *PtrNINV2* | Cytosol | Potri.013G006600.1 | 4449 | 2695 | 2007 | 668 | 75.81 | 5.68 | 179-645 | | |
|  |  | Potri.013G006600.2 | 4383 | 3003 | 1719 | 572 | 64.68 | 5.41 | 179-565 | | |
| *PtrNINV3* | Cytosol | Potri.008G024100.1 | 4586 | 2729 | 1992 | 663 | 74.26 | 6.31 | 174-640 | | |
|  |  | Potri.008G024100.2 | 4313 | 2565 | 1992 | 663 | 74.26 | 6.31 | 174-640 | | |
| *PtrNINV4* | Cytosol | Potri.010G236100.1 | 4633 | 2730 | 2001 | 666 | 74.71 | 5.93 | 176-642 | | |
|  |  | Potri.010G236100.2 | 4536 | 2249 | 1617 | 538 | 60.1 | 5.59 | 176-511 | | |
| *PtrNINV5* | Cytosol | Potri.005G010800.1 | 4486 | 2693 | 2001 | 666 | 76 | 5.89 | 168-643 | | |
|  |  | Potri.005G010800.2 | 4486 | 2666 | 1974 | 657 | 74.94 | 5.89 | 168-634 | | |
| *PtrNINV6* | Cytosol | Potri.004G186500.1 | 5863 | 2457 | 1857 | 618 | 69.66 | 7.82 | 130-598 | | |
|  |  | Potri.004G186500.2 | 5492 | 2455 | 1224 | 407 | 46.39 | 5.76 | 1-387 | | |
| *PtrNINV7* | Cytosol | Potri.005G239400.1 | 2504 | 1882 | 1668 | 555 | 63.51 | 6.14 | 85-546 | | |
| *PtrNINV8* | Cytosol | Potri.019G082000.1 | 6626 | 2215 | 1674 | 557 | 63.35 | 6.01 | 84-549 | | |
|  |  | Potri.019G082000.2 | 6594 | 2041 | 1674 | 557 | 63.35 | 6.01 | 84-549 | | |
|  |  | Potri.019G082000.3 | 6430 | 2103 | 1674 | 557 | 63.35 | 6.01 | 84-549 | | |
| *PtrNINV9* | Cytosol | Potri.004G167500.1 | 3632 | 2211 | 1722 | 573 | 65.43 | 6.16 | 103-563 | | |
| *PtrNINV10* | Cytosol | Potri.002G173600.1 | 4149 | 2169 | 2169 | 722 | 81.15 | 5.17 | 248-709 | | |
| *PtrNINV11* | Cytosol | Potri.009G129000.1 | 3372 | 2165 | 1725 | 574 | 65.67 | 5.93 | 104-564 | | |
| *PtrNINV12* | Cytosol | Potri.013G110800.1 | 7235 | 2268 | 1674 | 557 | 63.53 | 6.22 | 84-549 | | |
| *PtrNINV13* | Cytosol | Potri.014G100500.1 | 3096 | 702 | 702 | 233 | 26.17 | 8.95 | ***a*** | | |
| *PtrNINV14* | Cytosol | Potri.014G188200.1 | 520 | 520 | 387 | 128 | 14.66 | 6.55 | 5-111 | | |
| *PtrNINV15* | Cytosol | Potri.014G188100.1 | 928 | 928 | 216 | 71 | 8.31 | 8.03 | 2-34 | | |
| *PtrNINV16* | Cytosol | Potri.017G052800.1 | 201 | 201 | 201 | 66 | 7.95 | 9.57 | 1-59 | | |

***a***: indicate 23-61, 67-133, 140-173, 177-202, 204-232.
