# Supplementary material for: Genome-Wide Identification of the Invertase Gene Family in Populus
Source: PLoS One. 2015 Sep 22;10(9):e0138540. doi: 10.1371/journal.pone.0138540 (PMC4579127; doi:10.1371/journal.pone.0138540)
Supplement: S3 Table — (DOCX) [file pone.0138540.s004.docx]

**S3 Table. Coding region nucleotide (upper portion of matrix) and amino acid (bottom portion of matrix) sequence pairwise comparison (% identity) between poplar acid invertase sub-family genes.**

|  | *PtrCWINV1* | *PtrCWINV2* | *PtrCWINV3* | *PtrCWINV4* | *PtrCWINV5* | *PtrVINV1* | *PtrVINV2* | *PtrVINV3* |
| --- | --- | --- | --- | --- | --- | --- | --- | --- |
| *PtrCWINV1* | — | 87.61 | 85.8 | 58.05 | 56.46 | 50.51 | 46.46 | 47.84 |
| *PtrCWINV2* | 85.2 | — | 82.71 | 54.31 | 54.74 | 51.4 | 43.84 | 43.62 |
| *PtrCWINV3* | 85.2 | 80.75 | — | 58.79 | 57.38 | 50.83 | 47.31 | 47.44 |
| *PtrCWINV4* | 48.13 | 45.7 | 49.4 | — | 68.56 | 49.11 | 46.66 | 46.87 |
| *PtrCWINV5* | 48.38 | 45.75 | 50.68 | 61.55 | — | 49.1 | 45.84 | 46.3 |
| *PtrVINV1* | 42.05 | 40.42 | 43.81 | 43.03 | 42.61 | — | 69.88 | 55.88 |
| *PtrVINV2* | 37.46 | 35.41 | 39.67 | 39.28 | 38.27 | 69.49 | — | 62.2 |
| *PtrVINV3* | 37.83 | 34.5 | 39.17 | 38.25 | 37.48 | 51.94 | 55.96 | — |
