# Supplementary material for: Genome-Wide Identification of the Invertase Gene Family in Populus
Source: PLoS One. 2015 Sep 22;10(9):e0138540. doi: 10.1371/journal.pone.0138540 (PMC4579127; doi:10.1371/journal.pone.0138540)
Supplement: S4 Table — (DOCX) [file pone.0138540.s005.docx]

**S4 Table. Coding region nucleotide (upper portion of matrix) and amino acid (bottom portion of matrix) sequence pairwise comparison (% identity) between poplar neutral/alkaline invertase sub-family genes.**

|  | *PtrNINV1* | *PtrNINV2* | *PtrNINV3* | *PtrNINV4* | *PtrNINV5* | *PtrNINV6* | *PtrNINV7* | *PtrNINV8* | *PtrNINV9* | *PtrNINV10* | *PtrNINV11* | *PtrNINV12* |
| --- | --- | --- | --- | --- | --- | --- | --- | --- | --- | --- | --- | --- |
| *PtrNINV1* | — | 69.47 | 58.97 | 59.18 | 66.65 | 54.84 | 46.9 | 47.35 | 47.72 | 47.5 | 47.06 | 47.25 |
| *PtrNINV2* | 69.81 | — | 57.81 | 58.49 | 88.99 | 56.53 | 46.07 | 44.62 | 45.61 | 47.56 | 46.57 | 44.58 |
| *PtrNINV3* | 54.91 | 54.28 | — | 90.92 | 57.46 | 67.12 | 47.49 | 48.27 | 50.03 | 47.85 | 49 | 48.52 |
| *PtrNINV4* | 54.73 | 53.48 | 88.01 | — | 57.51 | 67.08 | 47.75 | 47.8 | 49.05 | 47.72 | 48.38 | 48.28 |
| *PtrNINV5* | 66.19 | 86.85 | 53.05 | 53.25 | — | 56.22 | 46.06 | 45.65 | 47.17 | 46.18 | 46.52 | 45.7 |
| *PtrNINV6* | 55.65 | 52.99 | 70.18 | 68.37 | 53.44 | — | 50.83 | 51.8 | 52.95 | 45.93 | 52.23 | 51.32 |
| *PtrNINV7* | 45.01 | 42.45 | 42.23 | 44.89 | 42.43 | 45.79 | — | 70.44 | 74.43 | 48.43 | 74.19 | 70.21 |
| *PtrNINV8* | 43.67 | 41.79 | 43.89 | 41.85 | 42.07 | 46.44 | 78.19 | — | 70.44 | 49.01 | 70.79 | 93.49 |
| *PtrNINV9* | 45.6 | 43.26 | 43.74 | 42.75 | 42.79 | 48.71 | 78.05 | 76.39 | — | 49.08 | 91.01 | 71.19 |
| *PtrNINV10* | 39.08 | 40 | 40.16 | 41.86 | 39.39 | 40.46 | 50.69 | 50.07 | 48.75 | — | 48.66 | 49.56 |
| *PtrNINV11* | 45.9 | 42.96 | 44.34 | 44.89 | 41.83 | 48.71 | 76.7 | 76.26 | 90.24 | 50.14 | — | 70.81 |
| *PtrNINV12* | 45.09 | 42.39 | 44.04 | 44.44 | 42.07 | 48.71 | 76.77 | 94.61 | 76.74 | 51.59 | 76.43 | — |
